# The full benefits of adult pneumococcal vaccination: A systematic review

**DOI:** 10.1371/journal.pone.0186903

**Published:** 2017-10-31

**Authors:** Elizabeth T. Cafiero-Fonseca, Andrew Stawasz, Sydney T. Johnson, Reiko Sato, David E. Bloom

**Affiliations:** 1 Data for Decisions, LLC, Waltham, Massachusetts, United States of America; 2 Performance Analysis and Improvement, Massachusetts General Hospital/Massachusetts General Physicians Organization, Boston, Massachusetts, United States of America; 3 Harvard Center for Population and Development Studies, Harvard T.H. Chan School of Public Health, Cambridge, Massachusetts, United States of America; 4 Global Health and Value, Pfizer Inc., Collegeville, Pennsylvania, United States of America; 5 Department of Global Health and Population, Harvard T.H. Chan School of Public Health, Boston, Massachusetts, United States of America; Public Health England, UNITED KINGDOM

## Abstract

**Background:**

Pneumococcal disease causes substantial morbidity and mortality, including among adults. Adult pneumococcal vaccines help to prevent these burdens, but they are underused. Accounting for the full benefits of adult pneumococcal vaccination may promote more rational resource allocation decisions with respect to adult pneumococcal vaccines.

**Objectives:**

Using the Preferred Reporting Items for Systematic Reviews and Meta-Analyses (PRISMA) guidelines, we conducted a systematic review to assess the extent to which the literature has empirically captured (e.g., through measurement or modeling) the full benefits of adult pneumococcal vaccination.

**Methods:**

We systematically searched PubMed and Embase to identify studies published between January 1, 2010 and April 10, 2016 that examine adult pneumococcal vaccination. We included articles if they captured any health or economic benefit of an adult pneumococcal vaccine administered to adults age ≥ 50 or ≥ 18 in risk groups. Finally, we summarized the literature by categorizing the types of benefits captured, the perspective taken, and the strength of the evidence presented. Our protocol is number 42016038335 in the PROSPERO International prospective register of systematic reviews.

**Results:**

We identified 5,857 papers and included 150 studies for analysis. While most capture health gains and healthcare cost savings, far fewer studies consider additional benefit categories, such as productivity gains. However, the studies with a broader approach still exhibit significant limitations; for example, many present only abstracts, while others offer no new measurements. Studies that examine the 13-valent pneumococcal conjugate vaccine focus more on broad economic benefits, but still have limitations.

**Conclusions:**

This review highlights the need for more robust empirical accounting of the full benefits of adult pneumococcal vaccination. Literature outside this realm indicates that these broad benefits may be substantial. Failing to investigate the full benefits may lead society to undervalue vaccines' contributions and therefore underinvest in their development and adoption.

## Introduction

Pneumococcal disease causes significant morbidity and mortality in both developing and developed countries, causing 1.6 million deaths annually [1]—more than seasonal influenza [2], malaria [3], or HIV/AIDS [4]. Pneumococcal disease comprises several clinical syndromes caused by the *Streptococcus pneumoniae* (pneumococcus) bacterium. Case fatality rates vary depending on the manifestation of the disease and can range from approximately 5% for pneumococcal pneumonia to 22% for adult pneumococcal meningitis. Certain pneumococcal infections, especially meningitis, can cause significant long-term sequelae [5].

The elderly and other adults at increased risk of contracting pneumococcal disease—including those with comorbidities or a compromised immune system—bear much of the burden of pneumococcal disease. Because of the potential for interpersonal disease transmission, older adults living in long-term care facilities are at a higher risk for contracting pneumococcal disease [6]. The incidence rate of pneumococcal disease increases with advancing age, and because the number of people age 60 years or older worldwide is expected to double between 2015 and 2050 (from 900 million to 2.1 billion) [7], pneumococcal disease in older adults will continue to be an important public health concern.

Two vaccines offer adults protection from pneumococcal disease: a 23-valent pneumococcal polysaccharide vaccine (PPV23) first introduced in 1983 [8] and a 13-valent pneumococcal conjugate vaccine (PCV13) first introduced in 2009 [9]. In Europe, PCV13 was initially approved for children from six weeks to five years of age in 2009 and then for adults age 50 years and older in 2011. The United States followed a similar pattern of introduction, approving the vaccine for infants and young children ages six weeks through five years in 2010, followed by approval for adults 50 and older at the end of 2011 [10].

While great strides have been made in pneumococcal vaccine distribution since their introduction, significant gaps in adult pneumococcal vaccine coverage persist. For example, in the United States in 2014, only 61.3% of adults 65 and older received their recommended pneumococcal vaccines, and coverage among high-risk adults age 19–64 (such as smokers or those with chronic conditions such as diabetes or chronic obstructive pulmonary disease) was only 20.3% [11]. Across Europe, recommendations and funding for adult pneumococcal vaccinations vary greatly in terms of age and risk groups [12, 13], making implementation and coverage less than optimal. Articulating and empirically measuring the full benefits of adult pneumococcal vaccination can help stimulate efforts to close coverage gaps and ensure adults receive appropriate protection from a vaccine-preventable disease.

### The value of vaccination

The finding that health promotes economic well-being—both individually and collectively—is a significant advance in the field of economic development [14–16]. It suggests that health interventions like vaccination programs have benefits that extend beyond the intrinsic value of mortality and morbidity reductions. Along these lines, recent research highlights how economists and policymakers have failed to account for vaccine programs’ full benefits [17–34]. This shortcoming suggests that these programs are substantially undervalued and that many decisions regarding vaccination adoption, scale-up, and investment in vaccine discovery and development were poorly founded.

Given global population aging [35] and the burden of pneumococcal disease among the elderly, adult pneumococcal vaccination in particular merits detailed analysis of the nature and economic magnitude of its potential benefits. This includes a critical review of existing research on the benefits of pneumococcal vaccines in adults. However, our approach differs from other such published systematic reviews in that we aim to determine the extent to which the literature captures broad benefits. Other reviews are typically restricted to a subset of benefits, such as economic benefits [36] or vaccine effectiveness [37–39].

We grounded this review in previous work that describes and attempts to understand the full benefits of vaccination [17, 19, 28, 30, 40–42]. As in other studies focusing on the broad benefits of particular vaccines [17, 28], we devised a taxonomy that identifies a comprehensive set of benefits of adult pneumococcal vaccination. The taxonomy distinguishes the narrow perspective, which includes benefits policymakers commonly think about, from a broader perspective, which includes additional benefits that policymakers rarely consider.

The following benefits constitute the narrow perspective and coincide with similar taxonomies for other diseases [17, 28]:

- Healthcare cost savings: The reduction in visits to medical practitioners, inpatient stays, and prescription drugs associated with pneumococcal disease treatment; and
- Health gains: The intrinsic value of reduced morbidity, mortality, pain, and suffering from pneumococcal disease.

Similarly, these additional benefits derive from existing benefit taxonomies for other diseases and reflect the broader perspective [17, 28]:

- Outcome-related productivity gains: The gains in productivity and income that accrue when immunized adults who are protected from pneumococcal disease are able to work and earn more;
- Care-related productivity gains: The value of caretakers’ productive time that is saved when they are released from the care and supervision of adults who are now healthier due to pneumococcal vaccination;
- Health-based community externalities: The value of improved health outcomes among nonvaccinated community members. These improved outcomes may be due to herd effects (to the extent that such effects are realized after adult pneumococcal vaccination) or due to a slowing of antibiotics’ loss of effectiveness, which imposes health and economic burdens; and
- Risk reduction gains: The value of pneumococcal vaccination’s role in reducing uncertainty and any concomitant anxiety, which would otherwise impose a cost on risk-averse people (e.g., value of peace of mind).

Other broad benefits are particularly relevant to pneumococcal vaccination, including:

- Voluntary contributions to family and community: The value of the human capital that accumulates in children and grandchildren through the greater time and education investments their healthier parents and grandparents make in them and the additional contributions adults make to their communities through participation in various activities, which they are better able to do when healthier;
- Prevention and amelioration of comorbidities: The inherent health benefits and further economic value of reduced incidence of comorbid health conditions (such as myocardial infarctions) and typical complications that follow from pneumococcal disease;
- Reduction in nosocomial infections: The value of avoiding nosocomial infections that could have followed from hospitalizations for pneumococcal disease had an adult not been vaccinated; and
- Promotion of social equity: The value of pneumococcal vaccination in diminishing social and economic inequalities, insofar as adult pneumococcal disease disproportionately affects the poor.

Using this taxonomy of benefits of adult pneumococcal vaccination as a guide, we systematically reviewed the literature to identify the breadth and depth of the evidence base on the benefits of adult pneumococcal vaccination.

## Methods

### Objectives

Our principal research objective was to survey the extent to which the literature empirically captures the full benefits of adult pneumococcal vaccination, through either direct measurement or modeling. Our secondary research objective, which fed into this central goal, was to survey the literature in terms of the breadth (i.e., kinds) of benefits captured and the strength and nature of evidence surrounding these benefits.

### Protocol and registration

The study protocol is registered on PROSPERO: International Prospective Register of Systematic Reviews and can be accessed at http://www.crd.york.ac.uk/PROSPERO/display_record.asp?ID=CRD42016038335 or viewed as supporting information S1 File. We conducted the review according to the Preferred Reporting Items for Systematic Reviews and Meta-Analyses (PRISMA) guidelines [43, 44].

To identify articles capturing the full benefits of adult pneumococcal vaccination, we performed a search on April 10, 2016 for the timeframe January 1, 2010 through April 10, 2016. We chose this timeframe because it captures a time during which both major adult pneumococcal vaccines were on the market. Although PCV13 had not been approved for older adults in Europe and the United States prior to 2011, we wanted to capture studies conducted to generate evidence on the potential benefits of adult pneumococcal vaccination, including modeling studies. We searched in two databases: PubMed and Embase. We used database terminology (Medical Subject Headings [MeSH] and Emtree, respectively) and keywords to capture more recent articles that had not yet been indexed in the database as of the search date. The search algorithm was based on two main topics: adult pneumococcal vaccination and benefits, which were subdivided into health benefits and economic benefits. S2 File presents the full search string, including the database terminology and keywords used. We exported the identified references into EndNote X7.5 and eliminated duplicate results. To check the sensitivity and quality of the search protocol, we identified 10 key articles that we felt the search should yield and confirmed that the search string returned all 10 articles. We also included articles from expert consultations to be as comprehensive as possible in the search.

Two reviewers screened the identified articles, first including or eliminating articles based on their titles and abstracts according to the inclusion and exclusion criteria that follow. Articles with ambiguous titles and abstracts underwent full text review by both reviewers. In the case the two reviewers disagreed, a third reviewer broke the tie. All reviewers were able to see the authors, institutions, journals of publication, and results when they applied the eligibility criteria. We recorded the reason for exclusion for studies excluded from full text review. To confirm that the reviewers applied the inclusion and exclusion criteria consistently, the two initial reviewers sorted 10 articles using these criteria. We reviewed discrepancies in the two reviewers’ judgments regarding these articles and discussed them to clarify the meaning of the criteria and to facilitate their consistent application.

We obtained most articles from Harvard, Tufts, and University of Massachusetts Boston libraries, or Google Scholar. We attempted to contact the authors of any articles unavailable from these sources and purchased articles that were still inaccessible when possible.

### Eligibility criteria

#### Inclusion criteria

According to our participants, interventions, comparators, outcomes, and study design (PICOS) criteria [43] (Table 1), we included articles in our review if they reported experimental, observational, or model-based studies that capture health or economic benefits of adult pneumococcal vaccination. These could include

- Economic evaluations of adult pneumococcal disease vaccination (including any method of analysis, such as cost-effectiveness analysis, cost-utility analysis, cost-minimization analysis, budget impact analysis, or benefit-cost analysis);
- Studies that calculate the benefit of any adult pneumococcal disease vaccination in health terms (e.g., quality-adjusted life years or QALYs, cases averted); or
- Studies that capture other benefits of adult pneumococcal vaccination that accrue to individuals or populations (e.g., reduction of hospitalizations due to pneumococcal pneumonia, reduction of nosocomial infections resulting from hospitalization due to pneumococcal disease).

**Table 1:** PICOS criteria for eligibility of studies.

PICOS Category	Description
Population	Vaccinated adults age 50 or above, or adults 18 and older in “risk groups” (as defined by the authors of the paper)
Interventions	Vaccination with PCV13 or PPV23
Comparator	Comparators as defined by the authors of the study, can include: • No vaccination; • Placebo; • Vaccination with another product (e.g., PCV13 rather than PPV23); • Co-administration of pneumococcal vaccine with influenza vaccine compared with vaccination with only one, or compared with no vaccination; and • Pneumococcal vaccination of a subgroup compared with another subgroup (e.g., immunocompromised adults compared with healthy adults)
Outcomes	Health or economic benefits of adult pneumococcal vaccination, including: • Healthcare cost savings; • Health gains; • Outcome-related productivity gains; • Care-related productivity gains; • Health-based community externalities; • Risk reduction gains; • Voluntary contributions to family and community; • Prevention and amelioration of comorbidities; • Reduction in nosocomial infections; and • Promotion of social equity
Study Design	Experimental, observational, or model-based studies that capture health or economic benefits of adult pneumococcal vaccination

Disease endpoints of interest included any form of adult pneumococcal disease, including pneumonia, meningitis, bacteremia, otitis media, sinusitis, bronchitis, ear infection, sinus infection, blood infection, and septicemia, or any comorbidities associated with pneumococcal disease. We included studies from any geographic area. We only included studies examining vaccinated adults age 50 or above, or adults 18 and older in “risk groups” (as defined by the authors of the paper; examples include adults with chronic illness, weakened immune systems, cochlear implants, or cerebrospinal fluid leaks, or adults living in long-term care facilities). We included studies of any pneumococcal vaccine product (as long as the vaccine is given to adults); any vaccination strategy; any length of follow-up; and, for economic studies, any currency.

#### Exclusion criteria

We excluded economic studies that did not compare costs with outcomes (e.g., price or cost studies, as opposed to, e.g., cost-benefit or cost-effectiveness). We excluded health studies relating solely to internal biological processes (e.g., antibody responses to vaccination) without directly connecting these processes to overall health or economic outcomes (e.g., disability-adjusted life years or DALYs, averted medical costs). We also excluded studies that only explore benefits stemming from vaccinating non-adults. We did not consider studies other than published articles, institutional reports (e.g., World Health Organization reports), and conference proceedings. We therefore excluded reviews, commentaries, editorials, news articles, and policy briefs. We excluded studies reported in any language other than English.

### Data extraction

We developed a template for extracting information from the included articles, pilot-tested it on a sample of five studies, and revised the template based on the pilot experience. Two reviewers divided the list of included articles alphabetically by the last name of the first author, extracted data according to the template, and conducted an audit on a subset of the included articles to quality-check the extraction. We extracted the following information from each included study:

- Study characteristics (study citation, control and treatment groups, limitations, assumed vaccination coverage, assumed duration of protection, time horizon of analysis, perspective taken, type of study, and follow-up time);
- Characteristics of the study population (setting, age group[s], and risk group[s] if applicable);
- Characteristics of the vaccination strategy (vaccine product[s] and vaccination strategy[ies]);
- Benefits captured in the study (type of outcome[s] measured, units, and results); and
- Contextual factors of interest (study sponsor or other comments on the analysis).

See S3 File for the template used to extract study information.

## Results

**Fig 1:**
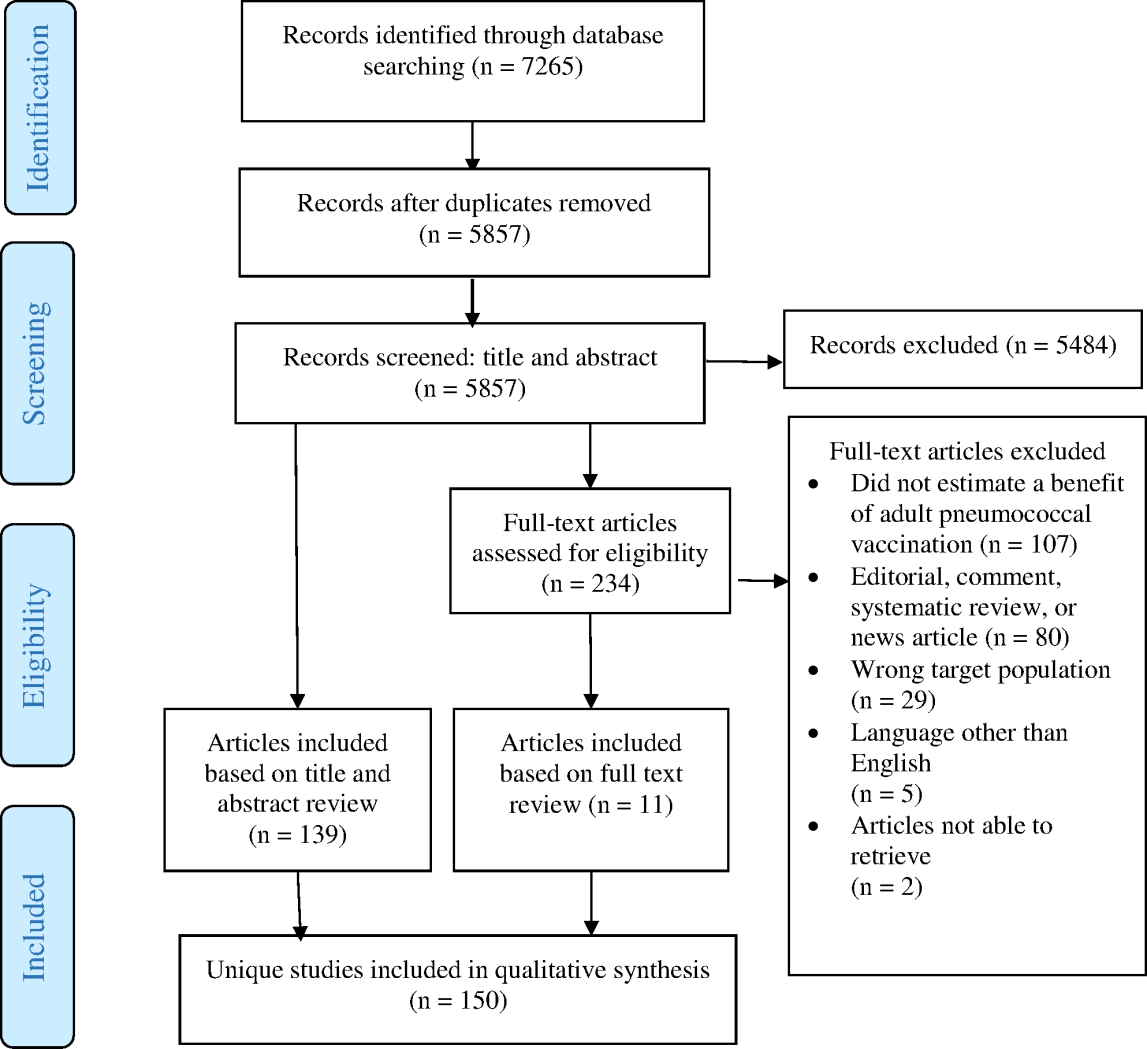
Flow diagram of study selection.

Application of the inclusion and exclusion criteria yielded 150 unique studies for analysis [45–194]. Fig 1 shows the yields at each stage of the review. Table 2 summarizes the reasons for excluding articles that reached the full text review stage. After attempting to access all the articles that reached the full text review stage, we were unable to obtain two articles and therefore excluded them from analysis [195, 196]. While the protocol allowed inclusion of articles recommended by experts during expert consultations and from snowball searching of references in included papers, we did not include any such articles, as our search captured all articles that either experts or reference lists brought to our attention.

**Table 2:** Summary of primary reasons for study exclusion after full text review.

Primary reason for exclusion	Number (%) of studies
Does not capture a health or economic benefit of pneumococcal vaccination	107 (47.98%)
Is a news article, comment, editorial, or review	80 (35.87%)
Examines a target population that does not meet our criteria (e.g., children)	29 (13.00%)
Is in a language other than English	5 (2.24%)
Could not access article	2 (0.90%)

S4 File contains the extracted information for all included articles. We could not to apply criteria to grade the quality of the evidence in the included studies because most studies captured in our review are modeling studies. Major published methods for assessing quality of evidence do not include modeling studies in their hierarchy [197]. In addition, some included articles are not modeling studies, meaning that methods of quality assessment would be difficult to apply in a consistent, meaningful manner. Furthermore, because of the varied nature of included studies, we were not able to assess risk of bias in a meaningful way.

### State of the literature

Table 3 summarizes the literature in terms of capturing the full benefits of adult pneumococcal vaccination. We found that, while the literature effectively captures narrow benefits, it rarely (if ever) captures all other benefits.

**Table 3:** Current state of the literature regarding the full benefits of adult pneumococcal vaccination.

Perspective		Benefit category	Brief description	Number (%) of studies that capture*
		Healthcare cost savings	Averted direct medical costs	79 studies (52.67%)
	Narrow	Health gains	Inherent value of improved health	149 studies (99.33%)
		Outcome-related productivity gains	Enhanced labor market output	16 studies (10.67%)
		Care-related productivity gains	Averted costs of formal or informal care	2 studies (1.33%)
		Voluntary contributions to family and community	Enhanced ability to volunteer and give care	0
Broad		Health-based community externalities	Herd effects or slowed pace of antimicrobial resistance	1 study (0.67%)
		Prevention and amelioration of comorbidities	Value of experiencing fewer or milder comorbidities	0
		Reduction in nosocomial infections	Averted hospital-acquired infection costs	0
		Risk reduction gains	Value of peace of mind from vaccines	0
		Promotion of social equity	Inherent value of narrowing health gaps	0

*This represents the number (and percent) of included studies that capture the benefit category in question; categories are not mutually exclusive.

#### Narrow benefits

Fifty-three percent of studies included in our systematic review included healthcare cost savings, though parameters used to estimate this dimension varied and were often based on estimates. Health gains were included in all but one study included for analysis. Many studies in the review account for this benefit directly. Others account for it indirectly by reporting QALYs or life years (LYs) lost to pneumococcal disease for cost-effectiveness or budget impact calculations.

#### Other broad benefits

The most commonly captured benefit from the broad perspective was outcome-related productivity gains. Sixteen studies (10.7%) included this benefit [74–76, 81, 85, 87, 98, 108, 115, 117, 127, 132, 142, 144, 157, 165]. These studies estimated how much work individuals miss due to a case of pneumococcal disease and compute the value of that missed work by multiplying missed time by the wage rate. At times, the amount of missed work is weighted by the proportion of the population that is economically active [74]. Two studies (1.3%) accounted for care-related productivity gains [74, 127]. One study (0.7%) accounted for health-based community externalities *vis-à-vis* the value of pneumococcal vaccination in slowing the rate of antimicrobial resistance [160]. Our included studies did not account for any other benefits in our taxonomy.

Even within the literature that examines benefits other than our narrow ones, study quality and reporting are at times limited. For example, of the 17 unique studies that account for at least one broad benefit, seven (41.2%) present only study abstracts [75, 81, 85, 87, 98, 115, 144]. Of the remaining 10 studies [74, 76, 108, 117, 127, 132, 142, 157, 160, 165], other limitations abound, including, for example, borrowing epidemiological trend data from other countries [108], lack of data necessitating many assumptions and low estimate precision [157], and possible confounding due to a study’s observational nature [160].

#### Perspectives assumed by economic studies

Examining the perspectives from which economic analyses were performed provides further insights into the state of the literature. Of the 75 included studies that explicitly stated a perspective, 51 (68.0%) examine costs from a health payer’s perspective, meaning they only consider direct costs (i.e., healthcare cost savings). Moreover, 24 studies claim to adopt a societal perspective (either as the only perspective studied or in addition to another perspective, such as health payer’s perspective), implying they consider other broad benefits [198]. However, this likely overstates the number of studies that look beyond narrow benefits. Some studies that were purportedly performed from the societal perspective appear only to consider narrow benefits. We therefore conclude that some studies’ perspectives may have been mislabeled.

Interestingly, three economic modeling studies—two from Brazil and one from Mexico—took an employer’s perspective to analyze the effects of vaccinating employees against pneumococcal disease on outcome-related productivity gains [81, 85, 98]. While the three studies presented only abstracts, and are therefore inconclusive by themselves, their findings unanimously favored the benefits of adult pneumococcal vaccination. This unanimity suggests that perspectives besides health payer might be worth exploring further.

#### Nature of PCV13 studies

Because PCV13 is the newest adult pneumococcal vaccine, isolating the included studies that analyze PCV13 alone is worthwhile. In general, the 32 (21.3%) studies that present an analysis of PCV13 alone tend to take a more economically focused approach than the other included studies. Twenty-seven (84.4%) of these PCV13-only studies account for healthcare cost savings [55, 63, 64, 80, 82, 87, 91, 98, 110, 117, 123, 127, 128, 130–132, 150, 151, 153, 156–158, 168, 173, 178, 184, 194], compared with 79 (52.7%) of all included studies. Similarly, seven of these studies (21.9%) account for outcome-related productivity gains [81, 87, 98, 117, 127, 132, 157], which represents double the share of all included studies (10.7%).

But PCV13-only studies have limitations. For example, a common limitation is that vaccine efficacy in adults is merely assumed for the purposes of economic modeling. This limitation was particularly common before the results of the CAPITA trial, a major randomized control trial of PCV13 in adults 65 and older, were published [57].

## Discussion

This systematic review shows that the literature does effectively capture some benefits of adult pneumococcal vaccination—notably, health gains and healthcare cost savings. To a limited extent, the literature takes a broader approach to estimating the benefits of adult pneumococcal vaccination. However, most benefits beyond the narrow approach are rarely, if ever, captured.

But this is not to say that these additional benefits are negligible. In fact, existing literature outside the scope of adult pneumococcal vaccines suggests that some benefits are potentially sizeable and are therefore worth investigating further.

The first of these potentially important benefits surrounds the notion that pneumococcal vaccines slow the rate of antimicrobial resistance (AMR) in at least two ways: by slowing the spread of especially resistant serotypes (such as serotype 19A, which is targeted by both commercially available adult vaccines) and by preventing illnesses and thus precluding the need for antibiotics. Although the literature on adult vaccination largely overlooks this effect, pneumococcal conjugate vaccines have been singled out as potentially significant players in the fight to reduce the rate of antibiotic resistance [199, 200]. This effect has clear community health benefits.

Second, recent evidence from the epidemiological literature suggests that some serotypes have low incidence among the young but high incidence among older adults [201]. Therefore, herd effects from vaccinating adults seem possible, which would spread the protective effects of these vaccines to nonvaccinated community members. This could be particularly important in settings with high concentrations of older adults, such as nursing homes. The strength of such herd effects would depend in large part on contextual factors such as childhood vaccine uptake rates [202]. Such factors should be accounted for when estimating the value of adult vaccination strategies.

Third, vaccinating adults attending mass gatherings may be an effective approach to limiting adult transmission [203]. Recognizing this, the Saudi Arabian government requires some vaccines for Hajj and Umrah pilgrims [204], including vaccines against meningitis and poliomyelitis. Including pneumococcal vaccination in the requirements for participants at mass gatherings could carry benefits for many parties: the vaccine recipient, other attendees who come into contact with vaccine recipients, travel companies that could offer the vaccine to appeal to risk-averse attendees, and government health ministries that would save money on treatment costs.

Fourth, parents and grandparents who are healthier are measurably better able to care for children and grandchildren [205, 206] and should in principle make greater voluntary contributions to their communities. Pneumococcal vaccines could contribute to these valuable ends.

Fifth, adults are demonstrably willing to pay to reduce risks to income [207] and to health [208]—both of which pneumococcal vaccines can help ameliorate.

Sixth, insofar as adult pneumococcal disease disproportionately affects the poor, vaccination can diminish social and economic inequalities [209, 210], an outcome that many consider inherently valuable.

Failing to investigate these and other sources of benefit further potentially leads to undervaluing adult pneumococcal vaccines' contributions and therefore underinvesting in their development and adoption. Additional work in this area should create an evidence base aimed at remedying these shortcomings.

### Limitations of this study

One limitation of our review is that we only included English-language studies. While we recognize that studies written in other languages likely contribute to the evidence base on the benefits of adult pneumococcal vaccination, our team’s shared language ability is limited to English. Another limitation of our study is the inability to apply a standard bias assessment to the included studies, such as the Cochrane Risk of Bias tool [197], which assesses risk of bias in randomized controlled trials, or ROBIS [211], a tool for assessing the risk of bias in systematic reviews. In lieu of a standard bias assessment, we recognize and comment on many of the limitations in the included studies (see S4 File). A third limitation of our analysis is that while many studies indicated a societal perspective, this was not clearly defined. Thus, we may have miscategorized some studies due to lack of information.

Finally, other vaccinations delivered in the same context, including influenza vaccination among target groups or pediatric vaccination programs, can affect the potential benefits of adult pneumococcal vaccination. This may make generalizing results to a different country context difficult. Several of our included studies demonstrate that co-administration of influenza and pneumococcal vaccination can have positive benefits [61, 62, 88, 164]. A recent systematic review and meta-analysis showed that high childhood vaccination coverage can lead to substantial herd effects, thus protecting unvaccinated adults from disease and reducing the magnitude of the potential benefits of adult vaccination [202]. The studies included in our review inconsistently account for herd effects from childhood vaccination (see S4 File for more detail). Therefore, any analysis of the full benefits of adult pneumococcal vaccination must consider this context.

## Conclusions

We conducted this systematic review to identify the breadth and depth of the benefits of adult pneumococcal vaccination captured in the literature. The review followed PRISMA guidelines (See S5 File). We included 150 unique studies for analysis. The literature effectively captures narrow benefits but rarely captures the full range of benefits in our framework. Further research is needed to quantify the broad benefits of adult pneumococcal vaccination.

## Supporting information

S1 File Systematic review protocol. **The Full Benefits of Adult Pneumococcal Vaccination: A Systematic Review**.
(PDF)

S2 File Appendix. **Search Terms. The Full Benefits of Adult Pneumococcal Vaccination: A Systematic Review**.
(PDF)

S3 File Appendix. **Extraction Template. The Full Benefits of Adult Pneumococcal Vaccination: A Systematic Review**.
(PDF)

S4 File Appendix. **Extraction Table. The Full Benefits of Adult Pneumococcal Vaccination: A Systematic Review**.
(XLSX)

S5 File Appendix. **PRISMA Checklist**.
(PDF)
