# Supplementary material for: The full benefits of adult pneumococcal vaccination: A systematic review
Source: PLoS One. 2017 Oct 31;12(10):e0186903. doi: 10.1371/journal.pone.0186903 (PMC5663403; doi:10.1371/journal.pone.0186903)
Supplement: S1 File — The Full Benefits of Adult Pneumococcal Vaccination: A Systematic Review. (PDF) [file pone.0186903.s001.pdf]

PROSPERO International prospective register of systematic reviews

The full benefits of adult pneumococcal vaccination: a systematic review

David Bloom, Elizabeth Fonseca, Sydney Johnson, Andrew Stawasz

Citation

David Bloom, Elizabeth Fonseca, Sydney Johnson, Andrew Stawasz. The full benefits of adult pneumococcal vaccination: a systematic review. PROSPERO 2016:CRD42016038335 Available from
http://www.crd.york.ac.uk/PROSPERO_REBRANDING/display_record.asp?ID=CRD42016038335

Review question(s)

Review Objective: Conduct a systematic literature review of studies that capture the health or economic benefits of adult pneumococcal vaccination.

What is the state of the literature in terms of capturing the full benefits of adult pneumococcal vaccination?

Of the benefits in the literature, what is known about each of the benefit categories?

- What is the evidence supporting t he benefit ?

- What is the magnitude of the benefit?

- How is the benefit distributed among different stakeholders?

Searches

We will search the following databases: PubMed and EMBASE. We will also include articles recommended by experts during expert consultations. We will use a combination of indexed terms (MeSH for PubMed and Emtree for EMBASE) and keyword terms.

We will restrict results to English. Studies published from 1/1/10 through the date of the search will be included. We will also include studies in any geographic area and evaluation of any pneumococcal vaccine product.

Types of study to be included

We will include both RCTs and observational studies.

Condition or domain being studied

Pneumococcal disease and its outcomes: (includes: pneumococcal infection, pneumonia, meningitis, invasive pneumococcal disease, non-invasive pneumococcal disease, bacteremia, otitis media, sinusitis, bronchitis, ear infection, sinus infection, blood infection, septicemia) or any pneumococcal disease comorbidities

Participants/ population

Inclusion: vaccinated adults age 50 or above or adults 18 and older in “risk groups” (risk groups as defined by authors of the paper)

Exclusion: studies that only explore benefits that stem from vaccinating non-adults (or risk groups)

Intervention(s), exposure(s)

"Adult pneumococcal vaccination" includes vaccination with any of the available pneumococcal vaccines, regardless of the delivery setting (e.g., health care setting, pharmacy).

Comparator(s)/ control

The control group in included studies can be a group of the same age that has not received vaccination against pneumococcal disease.

Context

All contexts are included (e.g., any country or geographic region; countries in which there are active childhood pneumococcal vaccination programs or not)

Outcome(s)

Primary outcomes

Health benefits of any adult pneumococcal disease vaccination (e.g., QALYs, cases averted)

Other benefits that accrue to individuals or populations when adults are vaccinated against pneumococcal disease related to reducing pneumococcal disease via adult vaccination (e.g., reduction of hospitalizations due to pneumococcal pneumonia, reduction of nosocomial infections resulting from hospitalization due to pneumococcal disease)

Secondary outcomes

None

Data extraction, (selection and coding)

Searches will be run in the three databases on the same day and results recorded in a flowchart (as recommended by the PRISMA Guidelines). Duplicate results will be eliminated (and the number of duplicates will be recorded for the flowchart).

Two reviewers will conduct the first round of review, including or eliminating articles based on title and abstract according to the inclusion/exclusion criteria. Articles that can't be categorized based on title/abstract will then undergo full text review by both authors. In the case of a discrepancy in the two authors' decisions, a third author will be consulted. The authors conducting the title/abstract and full text reviews will be able to see the authors, institutions, journal of publication and results when they apply the eligibility criteria. The number of identified references and those excluded will be recorded at each stage, including the reason for excluding any articles that reach the full text stage.

Two kinds of checks will be employed by the team:

1. Sensitivity of the search string: The authors have identified ten articles that the search should yield in order to check the sensitivity of our search string. If the articles are not returned by the search string, they will revise.

2. Ability of the criteria to be applied consistently by both reviewers: Ten articles will be pilot tested and run through the inclusion/exclusion criteria in order to compare the results from each reviewer. If there are discrepancies in the pilot test, the criteria can be clarified or refined so that they can be applied consistently. In addition, the number of contested articles will be recorded.

We will develop a data extraction sheet and pilot test it on a subset on included studies and refine it. Our initial list of data to extract includes:

• Study citation

• Year

• Vaccinated population

• Vaccine product

• Outcome category

• Specific outcome captured

• Result

• Unit(s) of measure

• Control and treatment groups (Vaccination and no-vaccination scenarios)

• Context of vaccination: Herd effects from childhood vaccination, conjunction with other vaccines (flu vaccine)

• Comments on the analysis

As with the initial review, two authors will extract the data, and a third author will act as a tie breaker as needed.

Risk of bias (quality) assessment
Two review authors will independently assess the risk of bias in included studies by considering the following characteristics that align with the Cochrane risk of bias tool:

For RCTs:

- was the randomization adequately generated?

- was the treatment adequately concealed/blinded from study participants and clinicians?

For both RCTs and Observational Studies:

- Were participant exclusions, attrition and incomplete outcome data adequately addressed in the published report?

- Selective outcome reporting: is there evidence of selective outcome reporting and might this have affected the study results?

- Was the study apparently free of any other sources of bias?

Strategy for data synthesis
We will provide a narrative synthesis of the findings of the systematic review. We will also provide counts of studies that capture different benefit categories from our taxonomy.

Analysis of subgroups or subsets
No planned analysis of subgroups or subsets.

Contact details for further information
Ms Fonseca

681 MAIN STREET, SUITE 3-37, WALTHAM, MA 02451

USA

?efonseca@datafordecisions.net

Organisational affiliation of the review
Data for Decisions, LLC and Pfizer Inc.

Review team
Professor David Bloom, Data for Decisions, LLC
Ms Elizabeth Fonseca, Data for Decisions, LLC
Ms Sydney Johnson, Data for Decisions, LLC
Mr Andrew Stawasz, Data for Decisions, LLC

Collaborators
Ms Reiko Sato, Pfizer, Inc

Anticipated or actual start date
01 April 2016

Anticipated completion date
31 December 2016

Funding sources/sponsors
Pfizer, Inc

Conflicts of interest
None known

Language
English

Country
United States of America

Subject index terms status
Subject indexing assigned by CRD

Subject index terms
Adult; Humans; Pneumococcal Vaccines; Streptococcus pneumoniae; Vaccination

Stage of review
Ongoing

Date of registration in PROSPERO
01 June 2016

Date of publication of this revision
01 June 2016

Stage of review at time of this submission	Started	Completed
Preliminary searches	Yes	Yes
Piloting of the study selection process	Yes	Yes
Formal screening of search results against eligibility criteria	Yes	No
Data extraction	No	No
Risk of bias (quality) assessment	No	No
Data analysis	No	No

PROSPERO
International prospective register of systematic reviews
The information in this record has been provided by the named contact for this review. CRD has accepted this information in good faith and registered the review in PROSPERO. CRD bears no responsibility or liability for the content of this registration record, any associated files or external websites.
