# Supplementary material for: The full benefits of adult pneumococcal vaccination: A systematic review
Source: PLoS One. 2017 Oct 31;12(10):e0186903. doi: 10.1371/journal.pone.0186903 (PMC5663403; doi:10.1371/journal.pone.0186903)
Supplement: S2 File — Search Terms. The Full Benefits of Adult Pneumococcal Vaccination: A Systematic Review. (PDF) [file pone.0186903.s002.pdf]

S2. File. Appendix. Search Terms: The Full Benefits of Adult Pneumococcal Vaccination: A Systematic Review

Database	Vocabulary	Theme	Search terms
**PubMed**	**MeSH**	Pneumococcal vaccination	"Pneumococcal Vaccines"[Mesh]
		Health benefits	"Severity of Illness Index"[Mesh] OR "Life Expectancy"[Mesh] OR "Longevity"[Mesh] OR "mortality"[Subheading] OR "Mortality"[Mesh] OR "Morbidity"[Mesh] OR "Survival Analysis"[Mesh] OR "Outcome Assessment (Healthcare)"[Mesh:NoExp] OR "Patient Outcome Assessment"[Mesh:NoExp] OR "Treatment Outcome"[Mesh] OR "Comorbidity"[Mesh] OR "Cross Infection"[Mesh] OR "Chronic Disease"[Mesh] OR "Activities of Daily Living"[Mesh] OR "Cardiovascular Diseases"[Mesh] OR "Immunity, Herd"[Mesh] OR "Depressive Disorder"[Mesh] OR "Depression"[Mesh] OR "Risk Reduction Behavior"[Mesh] OR "Hearing Disorders"[Mesh] OR "Paralysis"[Mesh] OR "Paresis"[Mesh] OR "Quality-Adjusted Life Years"[Mesh] OR "Quality of Life"[Mesh]
		Economic benefits	"Economics"[Mesh] OR "economics"[Subheading] OR "Models, Economic"[Mesh] OR "Hospitalization"[Mesh]
	**Full text**	Pneumococcal vaccination	pneumococcus vaccin*[tw] OR pneumococcal vaccin*[tw] OR pneumococcal conjugate[tw] OR pneumococcal polysaccharide vaccin*[tw] OR pneumococcal polysaccharide conjugate[tw] OR pneumococcal immuniz*[tw] OR pneumococcal immunis*[tw] OR pneumococcus immuniz*[tw] OR pneumococcus immunis*[tw] OR PCV7[tw] OR PCV13[tw] OR PCV10[tw] OR CVP[tw] OR PPV[tw] OR PPSV[tw] OR pneumovax[tw] OR synflorix[tw] OR prevnar[tw] OR prevenar[tw]
		Health benefits	vaccine impact*[tw] OR epidemiological change*[tw] OR comorbid*[tw] OR nosocomial[tw] OR cross infection*[tw] OR hospital infection*[tw] OR chronic disease*[tw] OR chronic illness*[tw] OR limitation of activity[tw] OR daily living[tw] OR cardiovascular disease*[tw] OR herd immunity[tw] OR depression[tw] OR

			depressive[tw] OR risk reduction*[tw] OR efficacy[tw] OR hearing[tw] OR deafness[tw] OR otitis media[tw] OR paralysis[tw] OR hemiparesis[tw] OR paresis[tw] OR paraparesis[tw] OR quality of life[tw] OR serotype replacement[tw] OR life expectanc*[tw] OR life year*[tw] OR mortality[tw] OR morbidity[tw] OR survival[tw] OR severity[tw] OR human capital[tw] OR burden*[tw] OR impact*[ti] OR impact*[ot] OR DALY*[tw] OR QALY*[tw] OR YLD[tw])
		Economic benefits	expenditure*[tw] OR healthcare cost*[tw] OR healthcare cost*[tw] OR hospitalization*[tw] OR hospitalisation*[tw] OR hospitalized[tw] OR hospitalized[tw] OR length of stay[tw] OR healthcare economic*[tw] OR healthcare economic*[tw] OR productivity[tw] OR wage*[tw] OR income*[tw] OR productivity[tw] OR cost-benefit[tw] OR benefit-cost[tw] OR cost effective*[tw] OR CEA[tw] OR ICER[tw] OR cost utility[tw] OR cost minimiz*[tw] OR treatment cost*[tw] OR health cost*[tw] OR medical care cost*[tw] OR cost control*[tw] OR cost analysis[tw] OR cost of healthcare[tw] OR cost of illness[tw] OR cost of disease[tw] OR high cost*[tw] OR budgetary impact*[tw] OR out of pocket[tw] OR pharmacoeconomic*[tw] OR economic evaluation*[tw] OR budget impact*[tw])
**Embase**	**Emtree**	Pneumococcal vaccination	'pneumococcus vaccine'/de
		Health benefits	'severity of illness index'/de OR 'longevity'/de OR 'survival'/exp OR 'mortality'/exp OR 'morbidity'/exp OR 'disease severity'/de OR 'disease duration'/de OR 'general condition deterioration'/de OR 'general condition improvement'/de OR 'poor general condition'/de OR 'deterioration'/de OR 'treatment outcome'/exp OR 'adverse outcome'/de OR 'drug efficacy'/de OR 'comorbidity'/de OR 'cross infection'/de OR 'hospital infection'/de OR 'chronic disease'/de OR 'daily life activity'/de OR 'cardiovascular disease'/exp OR 'herd immunity'/de OR 'depression'/exp OR 'risk reduction'/de OR 'hearing disorder'/exp OR 'hearing'/exp OR 'paralysis'/exp OR 'quality of life'/exp
		Economic benefits	'health economics'/exp OR 'statistical model'/de OR 'hospitalization'/de OR 'income'/de

	**Full text**	Pneumococcal vaccination	(pneumococc* NEXT/2 vaccin*):ab,ti OR (pneumococc* NEXT/2 conjugate):ab,ti OR (pneumococc* NEXT/2 immunis*):ab,ti OR (pneumococc* NEXT/2 immuniz*):ab,ti OR pcv7:ab,ti OR pcv13:ab,ti OR pvc10:ab,ti OR pneumovax:ab,ti OR synflorix:ab,ti OR prevnar:ab,ti OR prevenar:ab,ti OR ('pneumococcal infection'/exp OR 'bacteremia'/exp OR 'sinusitis'/exp OR 'sepsis'/de OR 'otitis media'/exp OR 'ear infection'/de OR bacteremia*:ab,ti OR sinusitis:ab,ti OR (sinus NEXT/1 infection*):ab,ti OR (blood NEXT/2 infection*):ab,ti OR (bloodstream NEXT/1 infection*):ab,ti OR septicemia:ab,ti OR septicaemia:ab,ti OR 'pneumococcal pneumonia':ab,ti OR (pneumococcal NEXT/1 infection*):ab,ti OR 'pneumococcal meningitis':ab,ti OR (ear NEXT/1 infection*):ab,ti OR 'otitis media':ab,ti OR (pneumococcal NEXT/1 disease*):ab,ti AND ('bacterial vaccine'/de OR 'immunization'/de OR 'mass immunization'/de OR 'vaccination'/de OR 'vaccine failure'/de OR vaccine:ab,ti OR vaccines:ab,ti OR vaccination*:ab,ti OR immunization:ab,ti OR immunisation:ab,ti))
		Health benefits	(vaccin* NEAR/2 impact*):ab,ti OR ((imminiz* OR immunis*) NEAR/3 impact*):ab,ti OR (epidemiolog* NEAR/1 change*):ab,ti OR comorbid*:ab,ti OR nosocomial:ab,ti OR (cross NEXT/1 infection*):ab,ti OR (hospital* NEAR/2 infection*):ab,ti OR (chronic NEXT/1 disease*):ab,ti OR (chronic NEXT/1 illness*):ab,ti OR 'limitation of activity':ab,ti OR 'daily living':ab,ti OR (cardiovascular NEXT/1 disease*):ab,ti OR (cardiac NEXT/1 disease*):ab,ti OR 'herd immunity':ab,ti OR depression:ab,ti OR depressive:ab,ti OR (risk NEAR/2 reduc*):ab,ti OR efficacy:ab,ti OR hearing:ab,ti OR deafness:ab,ti OR 'otitis media':ab,ti OR paralysis:ab,ti OR hemiparesis:ab,ti OR paresis:ab,ti OR paraparesis:ab,ti OR 'quality of life':ab,ti OR 'serotype replacement':ab,ti OR (life NEXT/1 expectanc*):ab,ti OR (life NEXT/1 year*):ab,ti OR mortality:ab,ti OR morbidity:ab,ti OR survival:ab,ti OR severity:ab,ti OR 'human capital':ab,ti OR burden*:ab,ti OR impact*:ti OR daly*:ab,ti OR qaly*:ab,ti OR yld:ab,ti
		Economic benefits	expenditure*:ab,ti OR ('healthcare' NEAR/3 economic*):ab,ti OR (healthcare NEAR/3 economic*):ab,ti OR (healthcare NEAR/2 cost*):ab,ti OR (health NEAR/2 cost*):ab,ti OR (treatment* NEAR/2 cost*):ab,ti OR (medical NEAR/2 cost*):ab,ti OR (cost*

			NEAR/2 illness):ab,ti OR (cost NEAR/2 disease):ab,ti OR 'cost benefit':ab,ti OR 'benefit cost':ab,ti OR (cost NEXT/1 effective*):ab,ti OR cea:ab,ti OR icer:ab,ti OR 'cost utility':ab,ti OR (cost* NEAR/1 minimiz*):ab,ti OR (cost* NEAR/1 control*):ab,ti OR 'cost analysis':ab,ti OR (high NEXT/1 cost*):ab,ti OR hospitalization*:ab,ti OR hospitalisation*:ab,ti OR hospitalized:ab,ti OR 'length of stay':ab,ti OR productivity:ab,ti OR wage*:ab,ti OR income*:ab,ti OR 'out of pocket':ab,ti OR pharmacoeconomic*:ab,ti OR (pharmaco NEXT/1 economic*):ab,ti OR (economic NEAR/2 evaluation*):ab,ti AND (budget* NEAR/2 impact*):ab,ti
