# Supplementary material for: The full benefits of adult pneumococcal vaccination: A systematic review
Source: PLoS One. 2017 Oct 31;12(10):e0186903. doi: 10.1371/journal.pone.0186903 (PMC5663403; doi:10.1371/journal.pone.0186903)
Supplement: S3 File — Extraction Template. The Full Benefits of Adult Pneumococcal Vaccination: A Systematic Review. (PDF) [file pone.0186903.s003.pdf]

S3. File. Appendix. Extraction Template: The Full Benefits of Adult Pneumococcal Vaccination: A Systematic Review

- Study citation,
- Setting,
- Studied population(s),
- Vaccine product(s),
- Vaccination strategy(ies),
- Outcome category(ies),
- Specific outcome(s) captured,
- Results,
- Unit(s) of measure,
- Control and treatment groups,
- Limitations,
- Assumed vaccination coverage,
- Assumed duration of protection,
- Time horizon of analysis,
- Perspective taken,
- Sponsor(s),
- Type of study, and
- Follow-up time.
